# Using large public data repositories to discover novel genetic mutations with prospective links to melanoma

**DOI:** 10.1186/1471-2105-16-S15-P3

**Published:** 2015-10-23

**Authors:** Tamas S Gal, Sally R Ellingson, Chi Wang, Jinpeng Liu, Stuart G Jarrett, John A D'Orazio

**Affiliations:** 1 Markey Cancer Center, University of Kentucky, Lexington, KY 40536, USA

## Background

Next generation sequencing (NGS) data analysis pipelines are frequently described in literature. NGS data is relatively easy to acquire from national data repositories and most software used in the pipelines are open source. This study extends research on the causal relation between changes in the ataxia telangiectasia and Rad3 related (ATR) pathways and melanoma[1].

## Materials and methods

**Figure 1:**
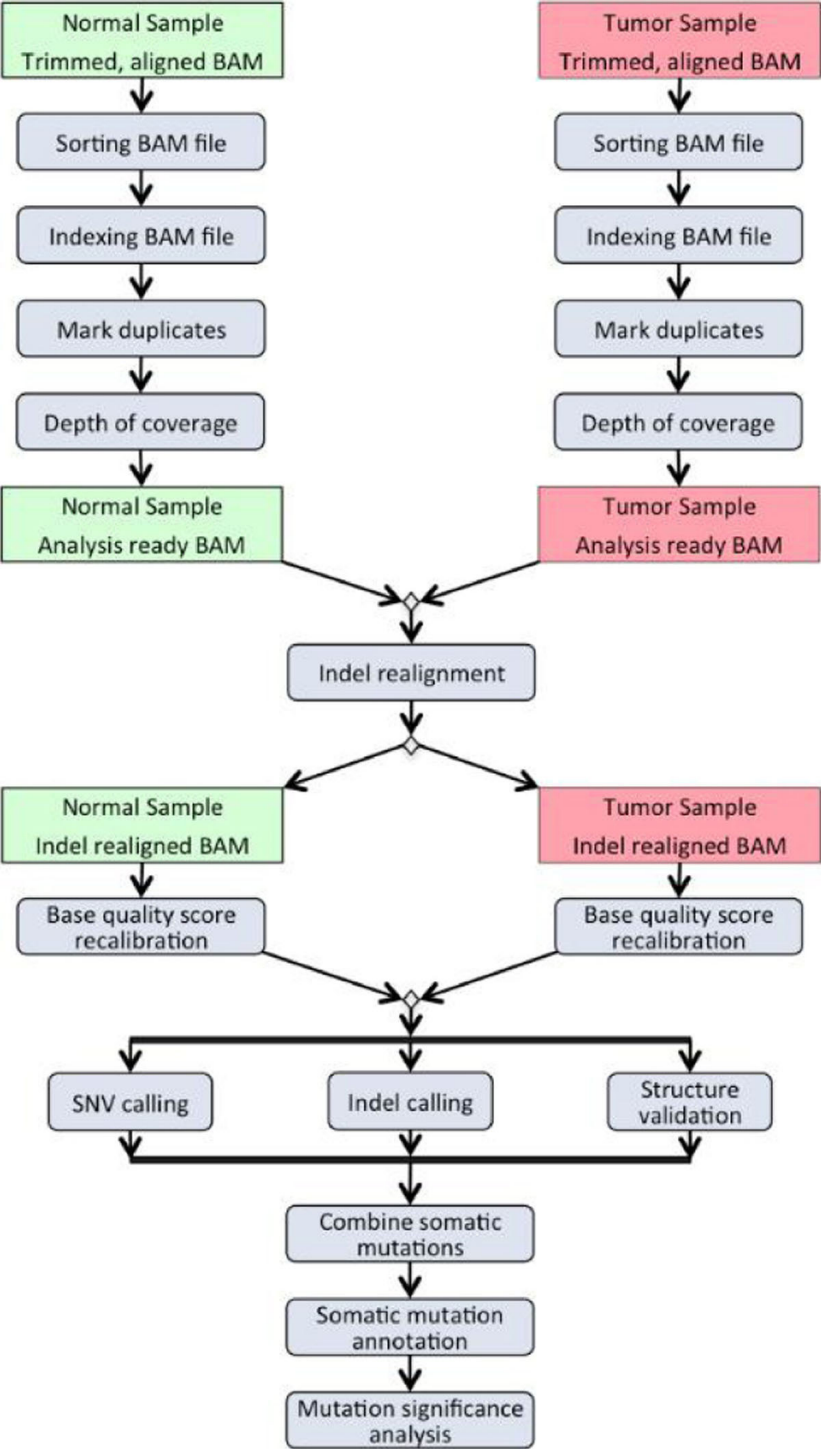
Pipeline for dbGaP analysis.

**Figure 2:**
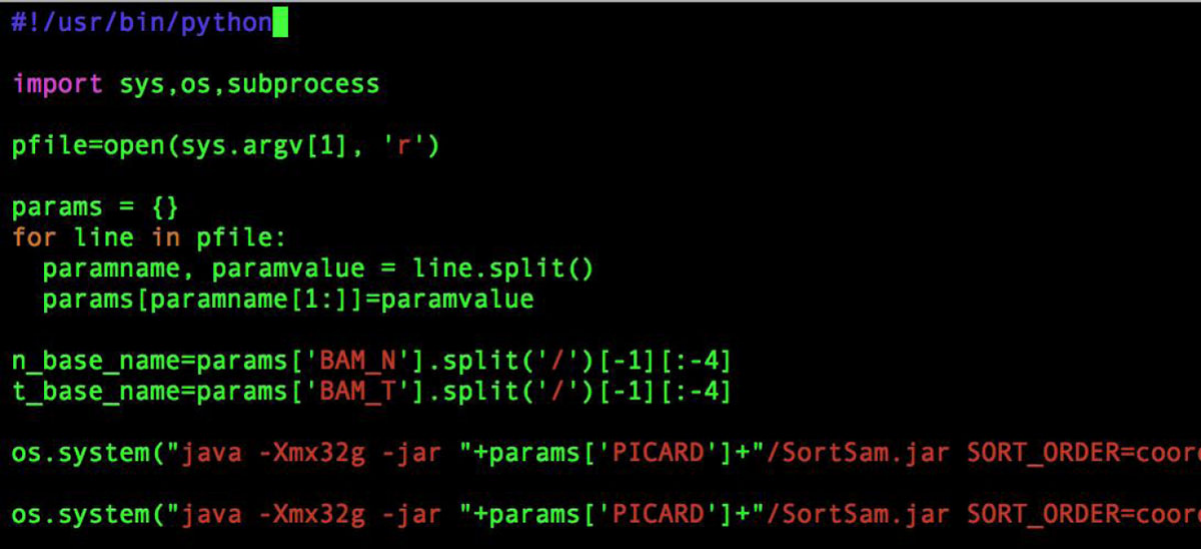
Python code for automated submission.

**Figure 3:**
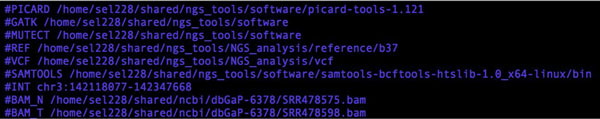
Configuration file.

To study the effects of mutations in the ATR region on melanoma, we downloaded the Melanoma Genome Sequencing Project dataset (dbGaP Study Accession: phs000452.v1.p1) from the dbGaP repository[2]. The dataset contained full exome sequencing data of paired normal and tumor samples of 122 phenotyped subjects in the format of trimmed and aligned BAM files. The dataset also included basic demographic information, such as gender and age; as well as disease specific variables, such as the localization of the melanoma and stage. The total size of the dataset was over 4TB, so we only downloaded the region of interest (ATR gene region) with 50Kbp padding before and after the ATR gene region. We used an available pipeline (Figure 1) for analysis that was previously developed for a lung cancer project by the Biostatistics and Bioinformatics Shared Resource Facility of the University of Kentucky Markey Cancer Center. The details of the data analysis pipeline will be published elsewhere. We used Python to automate data submission to the pipeline in combination with a configuration file that allowed us to easily swap different versions of the tools used in the pipeline and to match normal and tumor samples for the same patient (Figures 2, 3). Our experiments were executed on the Lipscomb High Performance Computing Cluster at the University of Kentucky.

## Results

Though analysis and validation of the results are still ongoing at the time of this report, we can share that we identified five previously unreported somatic missense or splice site SNP mutations in the ATR gene region in melanoma patients. Results will be further validated by analysis of NGS data from melanoma cell lines.

## Conclusions

The main goal of this abstract was to describe a methodology that we used to identify novel genetic markers in publicly available data. This methodology offers a cost effective way to test hypotheses drawn from laboratory research on human genome data.
